# Leveraging IsoArcH for isotope paleopathology: The example of the dataset from the Jedlička collection (Central Europe, 19th century CE)

**DOI:** 10.1016/j.dib.2023.109523

**Published:** 2023-09-22

**Authors:** Kévin Salesse, Sylva Drtikolová Kaupová, Arwa Kharobi, Antony Colombo, Jaroslav Brůžek, Vítězslav Kuželka, Petr Velemínský

**Affiliations:** a Department of Anthropology, Faculty of Science, Masaryk University, Kotlářská 2, 611 37 Brno, Czechia; b Department of Anthropology, National Museum, Václavské námĕstí 68, 11579, Praha 1, Czechia; c Department of Archaeology and Anthropology, Bournemouth University, Talbot Campus, Poole BH12 5BB, United Kingdom; d Ecole Pratique des Hautes Etudes-PSL University, Chair of Biological Anthropology Paul Broca, 4-14 rue Ferrus, F-75014 Paris, France; e UMR 6034 Archéosciences Bordeaux, Université Bordeaux Montaigne, F-33607 Pessac, cedex; f Laboratory of 3D Imaging and Analytical Methods, Department of Anthropology and Human Genetics, Viničná 7, 128 00 Prague 2, Czechia

**Keywords:** Stable isotope analysis, Carbon and nitrogen, Collagen, Bone trauma, Metabolic bone disease, Treponemal disease, Bone cancer, Bone infection

## Abstract

The article introduces the enhancements made to the IsoArcH database for isotope paleopathology. This includes the addition of new metadata fields, which allow for describing abnormal anatomical or physiological conditions in humans and animals at either the individual or sample level. To showcase the novel features of the database, the article features a unique dataset of carbon and nitrogen isotope values obtained on bulk bone collagen from 42 clinically-documented cases of the Jedlička pathological-anatomical reference collection, dating from the 19th century CE and curated at the National Museum in Prague, Czechia. The dataset includes 70 combined isotopic measurements from individuals who underwent anatomizations between 1841 and 1900 and had distinct bone diseases/disorders: *i.e.* syphilis, rickets, osteosarcoma, osteomyelitis, and healed fractures. Finally, the article highlights the value of the data in helping the isotope bioarchaeology and paleopathology communities in their understanding of disease processes.

Specifications Table

**Table:** 

Subject	Anthropology Archaeology
Specific subject area	*Isotope analysis* *Carbon* *Nitrogen* *Bone collagen* *Syphilis* *Bone fracture* *Rickets* *Osteosarcoma* *Osteomyelitis* *Paleopathology*
Type of data	Table Figure
How the data were acquired	Pre-treatments on bones were performed at the National Museum's Department of Anthropology located in Prague (Czechia). Bone powder underwent defatting using Kates' and Liden et al.'s methods [1,2]. Bone collagen was extracted using Longin's methodology [3], with modifications made by DeNiro and Epstein [4], Brown et al. [5], and Bocherens et al. [6,7]. Stable carbon and nitrogen isotopic compositions were determined using a Europa Scientific Roboprep elemental analyzer coupled with a Europa Scientific 20-20 isotope ratio monitoring mass spectrometer at Iso-Analytical Limited (Crewe, UK). Measurement accuracy and precision are reported following the guidelines outlined by Szpak et al. [8].
Data format	Raw
Description of data collection	Isotopic data were obtained from 42 individuals with various pathological conditions, such as syphilis, rickets, osteosarcoma, osteomyelitis, and healed fractures, sourced from Prague's pathological-anatomical reference collection. The dataset comprises 70 measurements of stable carbon and nitrogen isotope ratios conducted on bulk bone collagen samples.
Data source location	Material: Jedlička collection Institution: National Museum City: Prague Country: Czechia Geographic coordinates (WGS84): Lat 50.122014 N, Lng 14.629351 E
Data accessibility	Repository: IsoArcH (https://isoarch.eu/) [9,10] DOI of the dataset: 10.48530/isoarch.2023.002 Direct URL of the dataset: https://doi.org/10.48530/isoarch.2023.002 Data are available under the Creative Commons BY-NC-SA 4.0 license.
Related research article	Salesse, K., Kaupová, S., Brůžek, J., Kuželka, V., Velemínský, P., 2019. An isotopic case study of individuals with syphilis from the pathological-anatomical reference collection of the national museum in Prague (Czechia, 19th century A.D.), International Journal of Paleopathology 25, 46–55, https://doi.org/10.1016/j.ijpp.2019.04.001[11]

## 1 Value of the Data

• Pathological-anatomical reference collections showcasing bone diseases/disorders from the pre-antibiotic era are rare on a global scale, yet their value is tremendous, particularly in the context of isotopic studies. They offer a unique opportunity to provide insight into the extent to which bone pathologies, not influenced by modern drugs, influence the inherent stable isotope signatures in bone collagen. The present paper focuses on the Czech Jedlička collection.
• The article features carbon and nitrogen isotope data – most of which have never been published before – on five bone diseases/disorders that have received little or no attention in previous isotope studies. The potential value of this data lies in its ability to aid the isotope bioarchaeology and paleopathology communities in their understanding of disease processes.
• The IsoArcH database has been suitably adjusted to fulfill the requirements of isotope paleopathology and to integrate paleopathological data. The database is now a useful resource for paleopathologists and isotope researchers to share and initiate dialogue around paleopathological and isotopic data.

## 2 Objective

Isotope paleopathology is an emerging field that shows promise in shedding light on complex matters pertaining to human health [12]. In order to cope with the increasing amount of data and publications in this field, the IsoArcH database has been updated by adding new metadata fields related to paleopathological observations. The options to input free text for describing anomalous anatomical or physiological conditions is now available for both humans and animals, either at the individual or sample level. A one-of-a-kind dataset of carbon and nitrogen isotope values, specifically related to pathological materials extracted from the 19th century Jedlička collection curated at the National Museum in Prague, Czechia, is used as an example to showcase the novel features.

## 3 Data Description

**Fig. 1:**
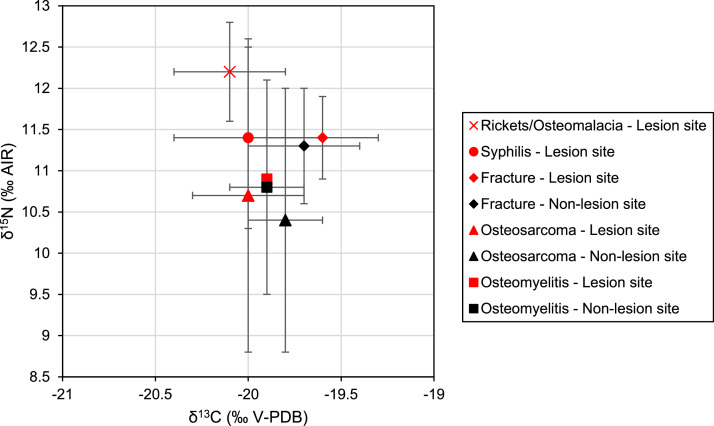
Biplot showing the mean and standard deviation of δ^13^C and δ^15^N values obtained from bone collagen samples of individuals with bone diseases/disorders from the Jedlička collection in Prague (19th century CE).

The present data article deals with materials deriving from a pathological-anatomical reference collection, the so-called Jedlička collection, curated at the the Horní Počernice's depositories of the Department of Anthropology in the National Museum in Prague (Czechia). Individuals with syphilis (*n* = 10), rickets (*n =* 7), osteosarcoma (*n =* 5), osteomyelitis (*n =* 10) and healed fractures (*n =* 10) were selected. Individuals afflicted by systemic diseases, such as rickets and syphilis, were sampled only once, with the exception of AJ3358/AJ3384, AJ3365/AJ3366 and AJ3369/AJ3370, for whom left and right femurs were sampled for testing intra-skeletal variations. Individuals afflicted by localized bone pathological changes or trauma were sampled twice, at the lesion site and distance to the lesion site. The final dataset consists of 70 combined measurements of stable carbon and nitrogen isotope ratios on bulk collagen. All but two measurements (at the lesion site for SA3374 and distant to the lesion site for OS2950) met the collagen quality criteria. These two measures are included in the dataset submitted to the IsoArcH database. The raw data are presented in Table 1. Means and standard deviations for each pathological condition are shown in Table 2 and Fig. 1. A subset of the dataset featured in this data article, comprising information on individuals who suffered from syphilis and had healed fractures, was released by Salesse et al. [11]. Plots were generated and calculations were conducted using Microsoft Excel.

**Table 1:** Biological and isotopic data of individuals from the Jedlička collection in Prague (19th century CE). Notes: The known age-at-death is mentioned in brackets. The given sources refer to the pages where the cases are described in Smrčka et al. [13]* and Ortner [14]**. %Col corresponds to the extraction yield, expressed as a weight percentage (wt.%). %C and %N indicates the carbon and nitrogen contents of samples and are expressed as percentages. δ^13^C and δ^15^N values were measured on bone collagen samples and are reported as per mil (‰) deviation relative to VPDB and AIR, respectively.

Individual ID	Date of anatomization	Biological sex	Age-at-death	Case ID	Bone disease/disorder	Sampled bone segment	Bone side	Sources	Sample ID	Sampled area	Distance between areas	%Col	%C	%N	C:N	δ^13^C	δ^15^N
1	1870	Female	Young adult [20]	AJ2303	Fracture/Rickets	Femur	Right		F-2303 Patho	Affected	26	29.4	43.5	15.8	3.2	−19.7	11.0
									F-2303 Well	Unaffected		31.9	43.1	15.5	3.2	−19.8	10.6
2	1881	Male	Middle adult [41]	AJ2863	Fracture	Femur	Right	p. 259*	F-2863 Patho	Affected	171	25.3	46.0	16.6	3.2	−19.1	11.8
									F-2863 Well	Unaffected		22.8	44.6	16.2	3.2	−19.5	11.4
3	1862	Male	Middle adult [49]	AJ2876	Fracture	Femur	Left		F-2876 Patho	Affected	137	24.4	42.3	15.5	3.2	−19.3	12.1
									F-2876 Well	Unaffected		21.6	41.7	15.2	3.2	−19.5	11.8
4	1871	Female	Adult [18+]	AJ2877	Fracture	Femur	Left	p. 261*	F-2877 Patho	Affected	114	24.5	43.6	16.0	3.2	−19.3	12.3
									F-2877 Well	Unaffected		23.1	41.2	15.0	3.2	−19.6	12.1
5	1883	Male	Young adult [23]	AJ3297	Fracture	Femur	Left	p. 278*	F-3297 Patho	Affected	127	24.1	45.4	16.5	3.2	−19.8	11.0
									F-3297 Well	Unaffected		22.7	44.7	16.4	3.2	−19.7	11.2
6	1847	Male	Young adult [22]	AJ3298	Fracture	Femur	Right	p. 285*	F-3298 Patho	Affected	125	28.6	42.9	14.9	3.4	−20.2	11.4
									F-3298 Well	Unaffected		23.4	42.3	15.4	3.2	−20.3	12.3
7	1888	Male	Old adult [52]	AJ3299	Fracture	Femur	Right		F-3299 Patho	Affected	74	23.9	44.8	16.1	3.2	−20.0	10.9
									F-3299 Well	Unaffected		22	45.0	16.5	3.2	−19.8	10.4
8	1865	Male	Middle adult [45]	AJ3300	Fracture	Femur	Right	p. 246*	F-3300 Patho	Affected	173	20.2	45.2	16.1	3.3	−19.7	11.0
									F-3300 Well	Unaffected		21.5	44.8	16.5	3.2	−19.9	10.5
9	1870	Female	Old adult [73]	AJ3306	Fracture	Femur	Right	p. 281*	F-3306 Patho	Affected	179	24.4	43.8	15.5	3.3	−19.7	11.4
									F-3306 Well	Unaffected		24.3	43.6	15.8	3.2	−19.5	11.5
10	1896	Male	Old adult [58]	AJ3312	Fracture	Femur	Right	p. 275*	F-3312 Patho	Affected	130	23.3	43.4	15.5	3.3	−19.5	10.8
									F-3312 Well	Unaffected		23	45.3	16.5	3.2	−19.5	11.0
11	[1850-1900]	Unknown	Adult [18+]	AJ2950	Osteomyelitis	Femur	Left	p. 188**	OS-2950 Patho	Affected	140	25.1	44.4	16.1	3.2	−20.1	9.6
									OS-2950 Well	Unaffected		24.4	49.7	17.8	3.3	−20.1	9.9
12	[1850-1900]	Female	Young adult [27]	AJ2994	Osteomyelitis	Tibia	Right	p. 386*	OS-2994 Patho	Affected	173	22	46.0	16.9	3.2	−19.9	11.8
									OS-2994 Well	Unaffected		22.8	44.4	16.3	3.2	−19.9	11.8
13	[1850-1900]	Male	Young adult [27]	AJ2995	Osteomyelitis	Tibia	Right	p. 518*	OS-2995 Patho	Affected	49	25.5	43.8	15.8	3.2	−19.5	11.1
									OS-2995 Well	Unaffected		23.9	43.0	15.8	3.2	−19.7	11.0
14	1872	Male	Adult [18+]	AJ2998	Osteomyelitis	Tibia	Left	p. 388*	OS-2998 Patho	Affected	30	24.4	34.5	12.0	3.4	−19.5	11.6
									OS-2998 Well	Unaffected		22.8	42.4	15.3	3.2	−19.5	11.1
15	1873	Female	Middle adult [40]	AJ3139	Osteomyelitis	Tibia	Left	p. 350*	OS-3139 Patho	Affected	130	23	32.1	11.4	3.3	−20.1	10.6
									OS-3139 Well	Unaffected		22.8	33.7	12.0	3.3	−20.0	10.6
16	1895	Female	Middle adult [40]	AJ3140	Osteomyelitis	Tibia	Right	p. 410*	OS-3140 Patho	Affected	191	23.2	36.0	12.8	3.3	−20.3	12.1
									OS-3140 Well	Unaffected		21.8	33.4	11.9	3.3	−20.2	12.3
17	[1850-1900]	Unknown	Juvenile [0-17]	AJ3144	Osteomyelitis	Femur	Right		OS-3144 Patho	Affected	250	23.8	33.5	12.0	3.3	−19.9	10.1
									OS-3144 Well	Unaffected		24.8	33.7	11.9	3.3	−19.9	10.3
18	[1850-1900]	Male	Young adult [25]	AJ3146	Osteomyelitis	Femur	Right	p. 301*	OS-3146 Patho	Affected	200	22	32.8	11.6	3.3	−19.8	9.2
									OS-3146 Well	Unaffected		22.7	34.0	12.1	3.3	−20.3	8.2
19	[1850-1900]	Unknown	Adult [18+]	AJ3148	Osteomyelitis	Femur	Left	p. 303*	OS-3148 Patho	Affected	165	23.2	34.4	12.1	3.3	−19.8	11.0
									OS-3148 Well	Unaffected		22.8	35.1	12.7	3.2	−19.9	9.5
20	[1850-1900]	Female	Middle adult [45]	AJ3273	Osteomyelitis	Tibia	Left	p. 392*	OS-3273 Patho	Affected	75	22.4	33.9	12.0	3.3	−19.7	12.3
									OS-3273 Well	Unaffected		24.1	33.4	11.7	3.3	−19.8	12.2
21	1871	Unknown	Adult [18+]	AJ3358	Rickets	Femur	Right	p. 317*	RA-3358	Affected		23	46.4	16.4	3.3	−20.0	11.9
				AJ3384	Rickets	Femur	Left	p. 317*	RA-3384	Affected		23.7	45.9	15.5	3.5	−19.9	11.3
22	[1850-1900]	Unknown	Adult [18+]	AJ3361	Rickets	Tibia	Right	p. 437*	RA-3361	Affected		26.3	44.6	15.1	3.4	−20.2	12.5
23	1870	Unknown	Adult [18+]	AJ3365	Rickets	Femur	Left	p. 315*	RA-3365	Affected		23.3	44.5	15.5	3.3	−20.1	12.0
				AJ3366	Rickets	Femur	Right	p. 315*	RA-3366	Affected		23.9	44.1	15.6	3.3	−19.9	11.3
24	[1850-1900]	Female	Old adult [80]	AJ3367	Rickets	Tibia	Left		RA-3367	Affected		25.3	44.9	15.0	3.5	−20.0	12.8
25	1841	Female	Adult [18+]	AJ3369	Rickets	Femur	Left	p. 502*	RA-3369	Affected		22.8	45.6	15.9	3.3	−20.0	13.3
				AJ3370	Rickets	Femur	Right	p. 502*	RA-3370	Affected		22.4	46.3	16.2	3.3	−19.8	13.0
26	1870	Unknown	Adult [18+]	AJ3371	Rickets	Femur	Left		RA-3371	Affected		23.5	43.5	14.8	3.4	−20.8	11.7
27	[1850-1900]	Male?	Adult [18+]	AJ3884	Rickets	Femur	Left	p. 320*	RA-3884	Affected		22.9	43.2	15.6	3.2	−19.7	11.8
28	1883	Male	Juvenile [17]	AJ2039	Osteosarcoma	Skull	–	p. 376**	SA-2039 Patho	Affected	105	25.7	44.2	15.4	3.3	−20.2	9.8
									SA-2039 Well	Unaffected		24.9	44.6	15.8	3.3	−20.1	9.6
29	1848	Female	Young adult [18]	AJ2044	Osteosarcoma	Skull	–		SA-2044 Patho	Affected	145	21.6	42.6	15.0	3.3	−20.0	13.3
									SA-2044 Well	Unaffected		23.9	43.6	16.4	3.1	−19.7	11.9
30	1889	Female	Old adult [54]	AJ2336	Osteosarcoma	Coxal bone	Left		SA-2336 Patho	Affected	110	25.1	43.6	16.1	3.2	−19.6	8.8
									SA-2336 Well	Unaffected		25.9	43.5	16.0	3.2	−19.7	8.7
31	1864	Female	Young adult [30]	AJ2964	Osteosarcoma	Femur	Right	p. 435*	SA-2964 Patho	Affected	260	20.7	43.8	15.6	3.3	−20.0	10.9
									SA-2964 Well	Unaffected		23.4	43.1	15.6	3.2	−19.7	12.3
32	1862	Male	Young adult [20]	AJ3374	Osteosarcoma	Tibia	Right	p. 430*	SA-3374 Patho	Affected	180	14	42.3	13.4	3.7	−20.9	11.1
									SA-3374 Well	Unaffected		23.4	42.9	15.7	3.2	−20.0	9.3
33	1891	Female	Young adult [31]	AJ2913	Syphilis	Tibia	Right	p. 427*	SY-2913	Affected		23.9	45.5	16.5	3.2	−20.0	11.2
34	1872	Female	Young adult [27]	AJ2914	Syphilis	Tibia	Left	p. 423* p. 285**	SY-2914	Affected		17.6	42.4	14.5	3.4	−19.9	11.0
35	[1850-1900]	Male?	Adult [18+]	AJ2918	Syphilis	Tibia	Left	p. 425*	SY-2918	Affected		24.6	45.3	16.1	3.3	−20.4	9.1
36	[1850-1900]	Unknown	Adult [18+]	AJ2943	Syphilis	Tibia	Right	p. 364*	SY-2943	Affected		23.4	44.8	15.9	3.3	−19.8	12.1
37	1879	Male	Old adult [54]	AJ2951	Syphilis	Tibia	–		SY-2951	Affected		24.6	45.5	15.3	3.5	−20.3	11.0
38	[1850-1900]	Unknown	Juvenile [15-17]	AJ3149	Syphilis	Tibia	–		SY-3149	Affected		26.3	43.7	15.8	3.2	−20.1	11.6
39	[1850-1900]	Unknown	Adult [18+]	AJ3155	Syphilis	Tibia	Left	p. 419*	SY-3155	Affected		24.3	45.9	16.1	3.3	−20.5	10.8
40	[1850-1900]	Unknown	Adult [18+]	AJ3162	Syphilis	Tibia	Right	p. 418*	SY-3162	Affected		24.1	44.7	16.3	3.2	−19.1	13.1
41	[1850-1900]	Unknown	Adult [18+]	AJ3173	Syphilis	Tibia	–		SY-3173	Affected		24.9	45.9	16.7	3.2	−19.9	12.2
42	[1850-1900]	Unknown	Adult [18+]	AJ3176	Syphilis	Tibia	–		SY-3176	Affected		25.4	44.5	16.0	3.2	−20.1	12.0

**Table 2:** Summary of isotopic data for individuals from the Jedlička collection in Prague (19th century CE). Notes: All measurements, except for data obtained at the lesion site for SA3374 and distant to the lesion site for OS2950 that did not meet the collagen quality criteria, are included in the table.

Bone disease/disorder	Sampled area	Number of individuals	δ^13^C	SD	δ^15^N	SD
Syphilis	Lesion site	10	−20	0.4	11.4	1.1
Osteosarcoma	Lesion site	4	−20	0.3	10.7	1.9
Osteosarcoma	Distant to the lesion site	5	−19.8	0.2	10.4	1.6
Rickets	Lesion site	7	−20.1	0.3	12.2	0.6
Osteomyelitis	Lesion site	10	−19.9	0.3	10.9	1.1
Osteomyelitis	Distant to the lesion site	9	−19.9	0.2	10.8	1.3
Fracture	Lesion site	10	−19.6	0.3	11.4	0.5
Fracture	Distant to the lesion site	10	−19.7	0.3	11.3	0.7

## 4 Experimental Design, Materials and Methods

### 4.1 Material and sampling strategy

The Jedlička collection comprises approximately 6,000 anatomical preparations of both soft and hard tissue diseases/disorders obtained from clinical human dissections performed between roughly 1840 and 1950. It includes unique examples of inflammatory and infectious bone diseases from the pre-antibiotic era, as well as numerous examples of skeletal abnormalities, bone disorders, and other osteological changes. Medical diagnoses were predominantly made on living patients, and clinical reports or documentations detailing the dissected cases have been preserved to this day for many of them [11,13]. The Jedlička collection is a rare, historical resource for the study of pathological anatomy and medicine, shedding light on population health and medical care in Czech lands and Central Europe during the 19th and 20th centuries CE.

A restricted quantity of samples was selected from the Jedlička collection, owing to its significance as a museum collection with historical importance, and the regulations in force at the National Museum in Prague to ensure its sustainability and continued preservation. A total of 45 bones from 42 individuals, who were afflicted by five different types of bone diseases/disorders, were selected (Table 1). For 33 of these instances, Ortner [14] and Smrčka et al. [13] published observations of the lesions and/or interpretations of the potential diseases (Table 1).

Bones with localized pathological changes related to osteosarcoma and osteomyelitis (from 5 and 10 individuals, respectively), and bone with healed fractures (from 10 individuals) were all sampled twice (Table 1). The intra-bone sampling procedure involved targeting the site of the lesion and an unaffected area located at a distance from the lesion on the same bone. This sampling strategy enables the examination of isotopic variations within the bone, providing the opportunity to investigate short-term changes in diet, physiological responses to pathological conditions and trauma, and/or the aftereffects of medical treatments. Individuals who had syphilis (*n =* 10), a systemic infection, or rickets (*n =* 7), a metabolic disorder, were not concerned by the multi-sampling process, and therefore, were sampled only once (Table 1). Bone fragments, ranging in size from 1 to 2 cm², were collected using a rotary tool.

Most bones sampled were femurs or tibias, though two skulls and one coxal bone were also sampled (Table 1). There are 29 individuals for whom the biological sex is reported. Specifically, there are 14 females and 15 males, with two of the male identifications being osteologically estimated (Table 1). Individuals are sorted into 5 distinct age groups, i.e. juvenile [0-17], adult [18+], young adult [18–35], middle adult [36–49], and old adult [50+] (Table 1). All the individuals under investigation have undergone anatomization between 1841 and 1900 (Table 1).

### 4.2 Bone collagen extraction for δ^13^C and δ^15^N analyses

Bone pretreatments were conducted at the Department of Anthropology of the National Museum in Prague. Bone sample was cleaned using a micro-drill equipped with a tungsten carbide drill bit to retain compact parts. Bone fragment was pulverized into a fine powder with the use of a ball mill, and particles ranging in size between 0.3 and 0.7 mm were collected.

Bone powder sample was defatted using the protocol outlined by Kates [1], and further validated by Liden et al. [2]. Powdered bone was soaked in 10 mL of a methanol-chloroform mixture (2:1, v/v) and subjected to ultrasonic treatment during 10 min. The supernatant containing lipids was discarded and the solution was renewed. This procedure was reiterated iteratively until full elimination of fatty acids and their derivatives was achieved. The sample was then rinsed thoroughly and oven-dried at 65°C for 7 h.

Bone collagen was extracted using the methodology described by Longin [3], with modifications made by DeNiro and Epstein [4], Brown et al. [5], and Bocherens et al. [6,7]. Bone powder sample (c. 200 mg) was demineralized in 20 mL of 1 M hydrochloric acid at room temperature for 20 min. Gelatin was collected using a Nalgene reusable filtration unit with Fisherbrand membranes (0.5 μm pores), washed, and then soaked in 20 mL of 0.125 M sodium hydroxide at room temperature for 20 h to eliminate fulvic and humic acids. The sample was filtered as previously described, and rinsed. Gelatin was subsequently solubilized in 15 mL of 0.01 M hydrochloric acid at 100 °C for 17 h and filtered using a 5–8 mm Ezee-filter to trap possible impurities. Finally, collagen samples were freeze-dried at −110 °C for a minimum of 48 h. The extraction yield (%Col), expressed as a weight percentage (wt.%), was determined at the conclusion of the procedure.

Bone collagen samples were analyzed for δ^13^C and δ^15^N using a Europa Scientific Roboprep elemental analyzer coupled with a Europa Scientific 20–20 isotope ratio monitoring mass spectrometer at Iso-Analytical Limited (Crewe, UK). Carbon and nitrogen contents of samples are expressed as percentages (%C and %N). Measurement error for both %C and %N, calculated from 14 duplicates of the international reference material IA-R042 (NBS-1577B bovine liver), was found to be ±2.49 (1σ) and ±0.55 (1σ), respectively. Weight percent carbon and nitrogen were used to calculate atomic C:N ratios. The δ^13^C and δ^15^N values were reported as per mil (‰) deviation relative to VPDB and AIR, respectively. Duplicate measurements were performed on 20 % of samples to test reproducibility (*n =* 15 samples, *i.e.* 30 measures). Measurement uncertainty was monitored using international and in-house standards with well-characterized isotopic compositions: IA-R042 (δ^13^C = −21.6 ± 0.06 ‰, δ^15^N = +7.65 ± 0.08 ‰), IA-R038 (L-alanine, δ^13^C = −24.99 ± 0.06 ‰, δ^15^N = −0.65 ± 0.04 ‰), IA-R006/IA-R046 (mixture of cane sugar and ammonium sulfate, δ^13^C = −11.64 ± 0.03 ‰, δ^15^N = +22.04 ± 0.06 ‰). Precision, *u(Rw)*, for both δ^13^C and δ^15^N, determined through repeated measurements of calibration standards, check standards, and sample replicates, was found to be ±0.08 ‰ and ±0.13 ‰, respectively. Systematic error, *u(bias)*, was determined to be ±0.14 for δ^13^C and ±0.13 for δ^15^N, based on the difference between observed and known δ values of the check standards and the long-term standard deviations of these standards. Total standard uncertainty was estimated to be ±0.16 ‰ for δ^13^C and ±0.18 ‰ for δ^15^N (Supplementary File 1). Measurement accuracy and precision are reported according to Szpak et al. [8], with calibration tables conveniently available on the dedicated IsoArcH landing page for the present dataset.

### 4.3 Collagen quality indicators

In modern bones, collagen extraction yields are found to be 20.4  ± 3.9 wt% (1σ) [7], and samples with less than 1 wt.% collagen are generally considered unreliable [15,16]. Carbon and nitrogen contents of modern bone typically range from 15.3 to 47 % and from 5.5 to 17.3 %, respectively [17]. Bone collagen samples with %C and %N below 13 % and 4.8 %, respectively, are generally regarded as significantly altered [11,16,17]. Atomic C:N ratios in modern bones are commonly around 3.2–3.3 [16,17], but can vary between 2.9 and 3.6 [18], with values outside these thresholds indicating potential alteration or contamination [11,17]. To maintain the high level of scientific rigor in the subsequent interpretative processes, it is strongly recommended to exclude any samples that fail to meet these criteria [19,20].

## Ethics Statements

This work does not involve any experimentation on living or modern-day human and animal subjects.

## CRediT authorship contribution statement

**Kévin Salesse:** Conceptualization, Methodology, Software, Validation, Formal analysis, Investigation, Data curation, Writing – original draft, Visualization, Funding acquisition. **Sylva Drtikolová Kaupová:** Methodology, Writing – review & editing, Funding acquisition. **Arwa Kharobi:** Writing – review & editing, Data curation. **Antony Colombo:** Writing – review & editing. **Jaroslav Brůžek:** Conceptualization, Writing – review & editing, Funding acquisition. **Vítězslav Kuželka:** Resources. **Petr Velemínský:** Conceptualization, Writing – review & editing, Project administration, Funding acquisition.

## Data Availability

Dataset of stable carbon and nitrogen isotope values from bone collagen samples of anatomized individuals from the Jedlička pathological-anatomical reference collection (Central Europe, 19th c. CE) (Original data) (IsoArcH Database)
